# 180. Multicenter Analysis of Adequacy of Empiric and Definitive Therapy by Time to Culture Result in Hospitalized Patients with Blood Culture Positive for Carbapenem Resistant and Susceptible Enterobacterales and *Pseudomonas aeruginosa*

**DOI:** 10.1093/ofid/ofad500.253

**Published:** 2023-11-27

**Authors:** Lauren Cooper, Kayla Van Benten, Kalvin Yu, ChinEn Ai, Anuprita Patkar, Sara Gregory, Vikas Gupta

**Affiliations:** Becton. Dickinson, Inc., Brownsburg, Indiana; Becton, Dickinson Inc., Fishers, Indiana; Becton, Dickinson and Company (BD), Franklin Lakes, New Jersey; BD - Becton, Dickinson and Company, Atlanta, Georgia; BD, Skillman, New Jersey; Springfield College, Glastonbury, Connecticut; Becton, Dickinson and Company (BD), Franklin Lakes, New Jersey

## Abstract

**Background:**

Rapid identification and antimicrobial susceptibility testing (AST) of pathogens causing bloodstream infections (BSI) is critical to providing accurate patient therapy. We conducted a multicenter evaluation of inadequate empiric (IET) and definitive therapy (IDT) from culture turn-around-time (TAT) in carbapenem susceptible (Carb-S) and carbapenem non-susceptible (Carb-NS) Enterobacterales (ENT) and *P. aeruginosa* (PSA) from blood specimens.

**Methods:**

Hospitalized adults (≥ 18 yo) with facility reported AST from 2018-2022 across 161 facilities in the BD Insights Research Database (Franklin Lakes, NJ) were identified for non-contaminant Carb-S and Carb-NS ENT and PSA from blood specimens. We evaluated antibacterial therapy as IET (from culture collection to prior to first AST result) and IDT (48-hours post first AST result and not discharged) by 12-hour increments of culture TAT (date/time first AST results minus date/time culture collection).

**Results:**

In total, we identified 42,125 ENT of which 1.2% (525) were Carb-NS, and 3,750 PSA of which 11.3% (423) were Carb-NS. The median time to AST results (hrs.) was significantly longer in Carb-NS ENT vs. Carb-S ENT (73.0 vs. 60.0) and in Carb-NS PSA vs. Carb-S PSA (77.8 vs. 69.5; **Table 1).** Average IDT was significantly lower than IET in Carb-S ENT (2.0% vs. 6.2%), Carb-NS ENT (15.1% vs. 45.6%), Carb-S PSA (3.6% vs. 10.8%) and Carb-NS PSA (14.6% vs. 39.8%) pathogen results. IDT was lower than IET at each 12-hour increment of availability of AST results (**Figures 2, 3**). IDT was also lower than IET at each 12-hour increment of availability of AST results for Carb-S ENT and Carb-NS ENT (**Figure 2**) and Carb-S PSA and Carb-NS PSA (**Figure 3).** Overall IDT was 15.1% and 14.6% in Carb-NS ENT and Carb-NS PSA, respectively, and only 2.0% and 3.6% in Carb-S ENT and Carb-S PSA, respectively.

**Table 1.**
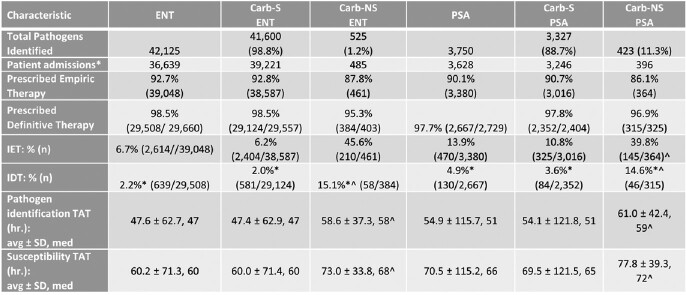
Characteristics for hospitalized adults with blood culture positive Enterobacterales (ENT) and P. aeruginosa (PSA). * p<0.0001 for IDT vs. IET; ^ p < 0.0001 for Carb-NS vs. Carb-S. Carb, carbapenem; IDT, inadequate definitive therapy (pathogen identified was not covered or was not susceptible to any antibiotics received from time of AST result until 48-hours later); IET, inadequate empiric therapy (pathogen identified not covered or was not susceptible to any antibiotics received from Day -2 until the time of AST result); NS, not susceptible; TAT, turn-around, time. * A patient’s admission can have both a Carb-S and Carb-NS pathogen.

**Figure 1.**
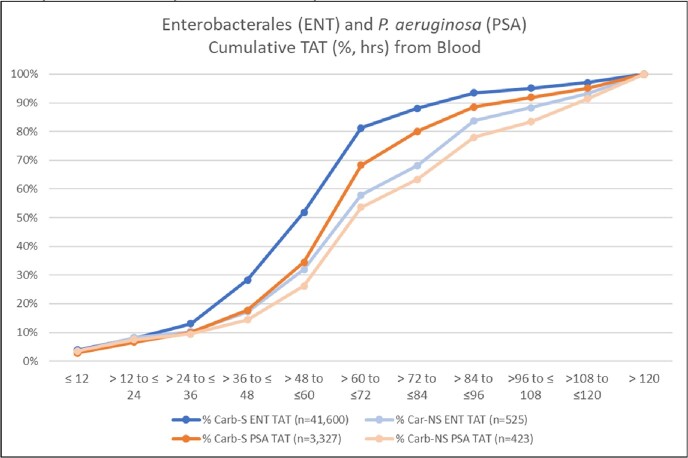
Cumulative TAT (hr.) for ENT, Carb-NS ENT, PSA and Carb-NS PSA in blood samples from hospitalized adult patients.

**Figure 2.**
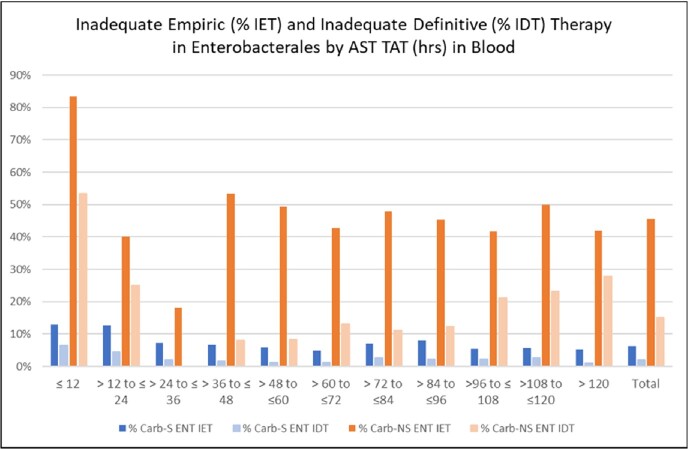
Percent IET and IDT in ENT and Carb-NS ENT by AST TAT (hours)

**Conclusion:**

The time it takes to obtain a pathogen identification and AST results for Carb-NS ENT and PSA organisms is longer than Carb-S ENT and PSA organisms, resulting in Carb-NS patients remaining on IET for extended periods of time. Additionally, since a higher percentage of Carb-NS patients never receive adequate targeted therapy, efforts to integrate diagnostic and antimicrobial stewardship can improve both timing of culture results and receipt of adequate therapy.

**Figure 3.**
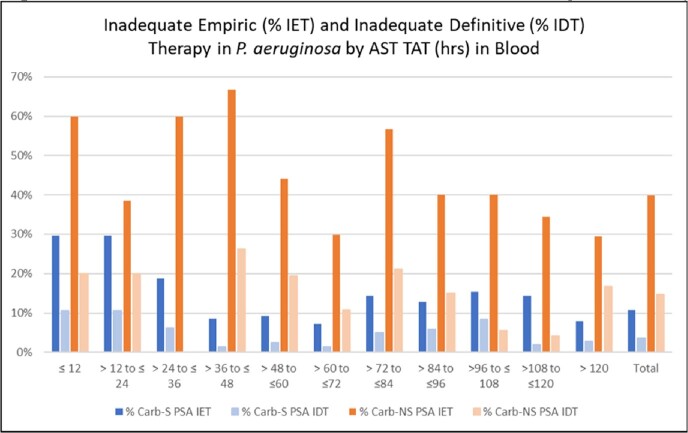
Percent IET and IDT in PSA and Carb-NS PSA by AST TAT (hours)

**Disclosures:**

**Lauren Cooper, PhD, MPH, D(ABMM)**, AbbVie: Grant/Research Support|Becton Dickinson, Inc: Stocks/Bonds **Kalvin Yu, MD, FIDSA**, BD: Stocks/Bonds **ChinEn Ai, MPH**, Becton, Dickinson and Company: Employee **Anuprita Patkar, PhD**, BD: Grant/Research Support|BD: Stocks/Bonds **Vikas Gupta, PharmD**, Becton, Dickinson and Company (BD): Employee of BD|Becton, Dickinson and Company (BD): Stocks/Bonds

